# Application and Development of Smart Thermally Conductive Fiber Materials

**DOI:** 10.3390/nano14020154

**Published:** 2024-01-10

**Authors:** Zhan Sun, Huitao Yu, Yiyu Feng, Wei Feng

**Affiliations:** Tianjin Key Laboratory of Composite and Functional Materials, School of Materials Science and Engineering, Tianjin University, Tianjin 300350, China; sunzhan810919@tju.edu.cn (Z.S.); huitaoy@tju.edu.cn (H.Y.); fengyiyu@tju.edu.cn (Y.F.)

**Keywords:** smart, thermally conductive fibers, personal thermal management

## Abstract

In recent years, with the rapid advancement in various high-tech technologies, efficient heat dissipation has become a key issue restricting the further development of high-power-density electronic devices and components. Concurrently, the demand for thermal comfort has increased; making effective personal thermal management a current research hotspot. There is a growing demand for thermally conductive materials that are diversified and specific. Therefore, smart thermally conductive fiber materials characterized by their high thermal conductivity and smart response properties have gained increasing attention. This review provides a comprehensive overview of emerging materials and approaches in the development of smart thermally conductive fiber materials. It categorizes them into composite thermally conductive fibers filled with high thermal conductivity fillers, electrically heated thermally conductive fiber materials, thermally radiative thermally conductive fiber materials, and phase change thermally conductive fiber materials. Finally, the challenges and opportunities faced by smart thermally conductive fiber materials are discussed and prospects for their future development are presented.

## 1. Introduction

**Figure 1:**
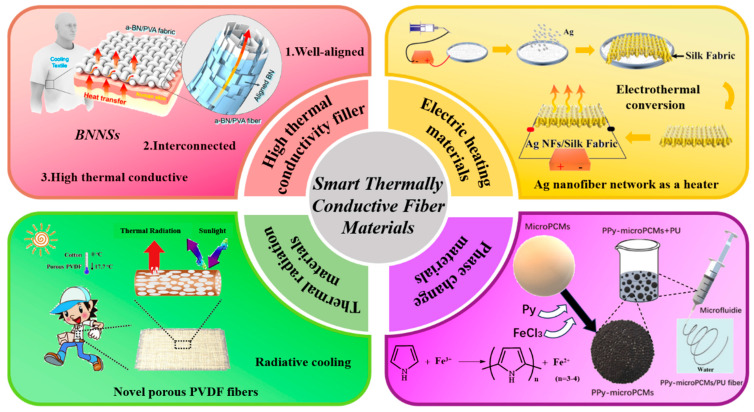
Classification of Smart Thermally Conductive Fiber Materials.

Recent advancements in space technology, artificial intelligence, green energy, and electronic communications have propelled the development of new information devices and military equipment toward miniaturization, integration, and increased power density [1,2,3]. This escalation in power density has resulted in increased heat accumulation within devices and localized surface heating. This poses significant challenges to the lifespan, performance, reliability, and safety of these devices [4,5,6,7,8,9]. According to statistics, from 2015 to 2021, China’s market for thermally conductive materials expanded from RMB 4.81 billion to RMB 15.62 billion; with growing applications in the military, aerospace, new energy automotive, and electronic information sectors [10]. The increasing diversification and specificity of people’s needs for thermally conductive materials present new requirements and challenges for traditional thermally conductive materials. Furthermore, the emphasis on energy conservation, the rising demand for personal temperature regulation, and the proliferation of wearable electronics and smart textiles have made effective personal thermal management (PTM) a critical area of research [11]. Consequently, developing thermally conductive materials that offer efficient thermal management has become a key research direction [12,13,14,15,16,17,18,19]. An increasing number of researchers are now focusing on smart thermally conductive fiber materials (Figure 1).

Smart thermally conductive fiber materials are distinguished by their high thermal conductivity (*λ*) and smart response characteristics for rapid and smart heat dissipation. These materials can adaptively regulate heat transfer in response to environmental changes or external stimuli. Their excellent thermal conductivity and ability to smartly adjust to specific requirements make them highly suitable for various applications. These include thermal management in electronic devices, smart body temperature regulation, and aerospace applications [20,21].

Compared with traditional thermally conductive materials, smart thermally conductive fiber materials offer several advantages:

(1) These materials possess a more complex structure and composition. Traditional thermally conductive materials, such as metal plates and ceramics, typically have a homogeneous and continuous structure. However, based on interdisciplinary collaboration and technological advancements in materials science, information technology, biology, and other disciplines, it is possible to prepare green, super-performance, smart thermally conductive fiber materials with multicomponent, multistructured, and multifunctional properties [22]. This enables the integration and optimization of performance by providing a larger surface area and better heat conduction paths. This enhances their *λ*.
(2) Smart thermally conductive fiber materials exhibit greater plasticity and adaptability. These smart materials can be designed in terms of dimensions, structures, and components to meet diverse environmental and application-specific requirements. Their plasticity and adaptability surpass those of traditional materials, which often have rigid shapes and structures that limit their versatility.
(3) Smart thermally conductive fiber materials possess superior functional characteristics. It is possible to design the fibers to achieve more specific and efficient thermal management functions for different application scenarios. For instance, phase change thermally conductive fibers can absorb and release heat through phase change energy storage, providing temperature regulation and control. Similarly, carbon nanotube thermally conductive fibers, which are known for their higher thermal conductivity, facilitate more efficient heat transfer [23].
(4) Smart thermally conductive fiber materials have a wider range of applications. These materials have broader applications than their traditional counterparts; notably, in clothing and electronic device heat dissipation. For example, Fan et al. developed a new all-weather personal thermal management textile called TAWT, which provides advanced ideas and methods for the development of multifunctional textiles [24].

Smart thermally conductive fiber materials can make a big difference in our daily lives. For example, this type of material can be used in clothing for those in services exposed to excessive temperature differences, such as the fire brigade. As we all know, coveralls are one of the most important pieces of equipment to protect the personal safety of firefighters who are active on the first line of firefighting, and they are an indispensable necessity at fire rescue sites. The integration of smart thermally conductive fiber materials into firefighting coveralls allows for intelligent thermal management for firefighters, and enables them to maintain as much thermal comfort as possible, even in extreme environments.

This review introduces the concepts of thermal conductivity, fibers’ thermal conductivity characterization, and PTM. It then delves into the discussion of emerging thermally conductive fiber materials and their applications based on various heat transfer mechanisms. Finally, it summarizes the key challenges in the research of smart thermally conductive fiber materials and predicts future development trends and their important potential applications in many fields. This provides new perspectives and strategies for the design and creation of innovative thermally conductive fiber materials.

## 2. Basic Concept

Thermal conductivity is a fundamental concept in heat transfer, which refers to the transfer of thermal energy from a higher-temperature region to a lower-temperature region. Thermal conductivity is based on the interaction and motion of molecules within solids, liquids, and gases. The mechanisms of thermal conduction vary among different substances, including conductive, convective, and radiative heat transfer. Recent advancements in smart thermal conduction have introduced innovative strategies, such as the introduction of phase change materials and biomimetic optimization. Biomimetic optimization involves designing structures exhibiting excellent thermal conduction inspired by nature. For example, thermal conduction channels with a porous structure can be designed to emulate a honeycomb structure in order to increase the surface area for heat conduction. Similarly, the streamlined body structure of fish can be imitated to reduce fluid resistance in thermal conduction channels and enhance thermal conduction efficiency. These novel strategies offer new insights and approaches for smart thermally conductive fiber development. The key parameters defining *λ* of the fibers include thermal conductivity, specific heat capacity, thermal resistance, and thermal diffusion coefficient. Thermal conductivity and thermal resistance are inversely related performance indicators, and a woven fabric with small thermal conductivity or large thermal resistance is observed to be well insulated. Due to the arrangement of molecules in the fiber, there is an axial orientation [25]; this results in different thermal conductivities along the radial and axial directions of the fiber. This means that there is anisotropy in the *λ* of the fibers. In general, the axial thermal conductivity of fibers is considerably greater than the radial thermal conductivity. For example, the *λ* of nylon fibers is 0.244–0.337 W m^−1^ K^−1^, while *λ*_||_ is 0.5934 W m^−1^ K^−1^, and *λ*_⊥_ is 0.2701 W m^−1^ K^−1^ [26].

To understand the concept of PTM, it is important to first understand thermal comfort. Thermal comfort is defined by Hensen as “the psychological state of being satisfied with the thermal environment” [27]. It is a type of psychological satisfaction influenced by various factors, including physical, psychological, and physiological elements [28]. It can be straightforwardly described as follows: when the human body is in a state of thermal equilibrium, the skin’s temperature is close to the standard body temperature. The absence of thermal comfort may result in decreased work efficiency and various health problems [29]; thermoregulation of the human body is, therefore, indispensable. PTM technology is recognized as an emerging strategy to enable personalized and energy-efficient control of human thermoregulation. Its objective is to supply the appropriate amount of heat to individuals to maintain optimal thermal comfort [30,31]. PTM technologies, which encompass personal cooling, heating, insulation, and thermoregulation, offer a more adaptable and comprehensive solution than traditional air/liquid cooling methods. In the past decade, the development of novel advanced materials and strategies has considerably enhanced the performance and comfort of PTMs, and their future development prospects are very promising [32,33,34,35,36].

## 3. Advanced Thermally Conductive Fiber Materials

### 3.1. Composite Thermally Conductive Fibers with High Thermal Conductivity Fillers

As a primary method for dissipating heat from electronic devices, thermal conduction is a key factor to improve heat dissipation and is an important research element to achieve efficient thermal management. Meanwhile, for infrared opaque materials, heat conduction is the main heat transfer pathway at the interface between human skin and the inner surface of the textile materials. Moreover, thermal conduction is the only pathway for heat dissipation within fibrous materials. Therefore, the design and development of highly thermally conductive fibers with modulated properties is of great value in achieving efficient thermal management.

**Figure 2:**
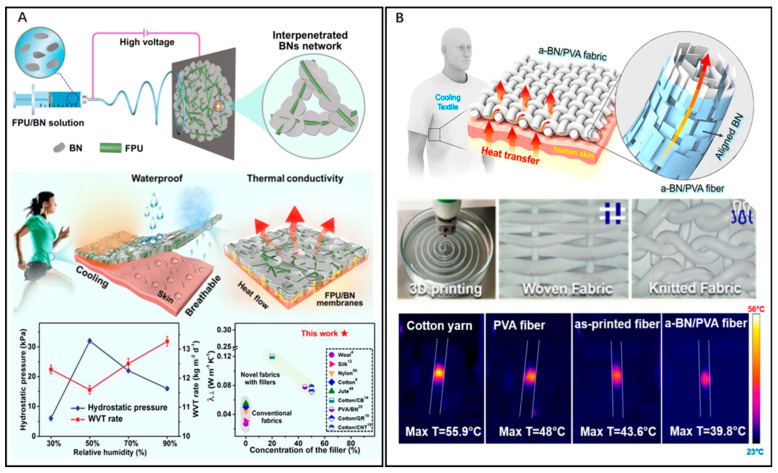
(**A**) Schematic representation of the preparation and structure of FPU/BN membranes; schematic representation of waterproofing, air permeability and thermal conductivity of FPU/BN membranes; thermal conductivity of this as well as other notable published studies on thermally conductive textiles; Rct and Ret of FPU/BN films prepared at different relative humidities. Reprinted with permission from Ref. [43]. Copyright 2020 American Chemical Society. (**B**) Schematic diagram of a-BN/PVA thermally conditioned textile; preparation of a-BN/PVA fibers using 3D printing; different structures of a-BN/PVA textiles can be observed in optical images: woven and knitted fabrics in plain weave; temperature distribution in cotton yarn, PVA fiber, a-BN/PVA fiber, and as-printed BN/PVA fiber without BNNS alignment. Reprinted with permission from Ref. [22]. Copyright 2017 American Chemical Society.

In order to achieve heat transfer, the addition of fillers with great thermal conductivity such as carbon nanotubes (CNTs) [37], graphene [38], boron nitride (BN) [39,40,41], and transition metal carbide/nitride (MXene) [42] can be integrated into conventional fibers to increase the *λ* of the fibers to achieve temperature reduction. This type of composite fiber provides a more practical and cost-effective approach for thermal management. Among them, BN nanosheets are the most favorable fillers for engineered thermally conductive fibers due to their extremely high thermal conductivity, exceptional chemical stability, affordable price, and strong affinity for colored dyes. Over the past decades, researchers have attempted to enhance the *λ* of fibers by combining fibers and BN nanosheets using various processing techniques such as dip coating, wet spinning, and 3D printing. As shown in Figure 2A, Yu et al. [43] blended hydrophobic fluorinated polyurethane (FPU) and high thermal conductivity boron nitride (BN) nanosheets using a one-step electrostatic spinning method, and controlling the loading rate of boron nitride nanosheets and the relative humidity (RH) of the environment to create a good BN interpenetrating network. This method allowed the BN to interconnect along the nanofibers while the porous structure of the material was maintained; this enhanced thermal conductivity while maintaining moisture permeability. Due to the extremely minimal surface energy of fluorinated polyurethane, its incorporation can effectively enhance the hydrophobicity of the fibers. The final prepared fibrous membranes have excellent active and passive cooling properties and outstanding water resistance. It has an ultra-high within-plane *λ* of 17.9 W m^−1^ K^−1^, a cross-face *λ* of 0.29 W m^−1^ K^−1^, and a high water vapor transmission rate (WVT) of 11.6 kg m^−2^ day ^−1^. In addition, it exhibits excellent water resistance, as evidenced by its high water contact angle of 153° and its ability to withstand a static water pressure of 32 kPa. This makes it a strong candidate for next-generation cooling fiber textiles.

Gao et al. [22] developed a thermally conductive fiber textile for PTM, as shown in Figure 2B. They synthesized a well-aligned boron nitride (BN)/polyvinyl alcohol (PVA) composite fiber using 3D printing technology, which greatly improved the thermal transfer properties of the fiber. BN, as a two-dimensional anisotropic material, has a high within-plane *λ* of 2000 W m^−1^ K^−1^ and a low vertical *λ* of 20–40 W m^−1^ K^−1^. It is widely used in thermal management applications [44,45,46,47]. The authors first placed boron nitride nanosheets (BNNS) in PVA solution for ultrasonication to make them homogeneously dispersed. Then, through uniaxial tensile flow during fiber printing and further stretching processing, BNNS are highly aligned on the composite nanofibers to form a boron nitride (BN)/polyvinyl alcohol (PVA) composite fiber (referred to as a-BN/PVA). The fibers exhibit high thermal conductivity, which enables the transfer of body heat to the surrounding environment through the textile fibers. Compared with cotton textiles, the new textile has more than twice the *λ* of the former. The a-BN/PVA fiber is woven into a wearable textile, which can effectively transfer heat from the human body to the exterior of the fabric fibers, thereby achieving a thermal cooling effect and personal thermal management. Further mechanical property tests and thermal tests have verified its wear resistance and thermal conductivity [48,49]. The wearable a-BN/PVA fiber textile prepared using 3D printing technology can significantly reduce the cost of cooling and provides a promising option for personal cooling needs.

**Figure 3:**
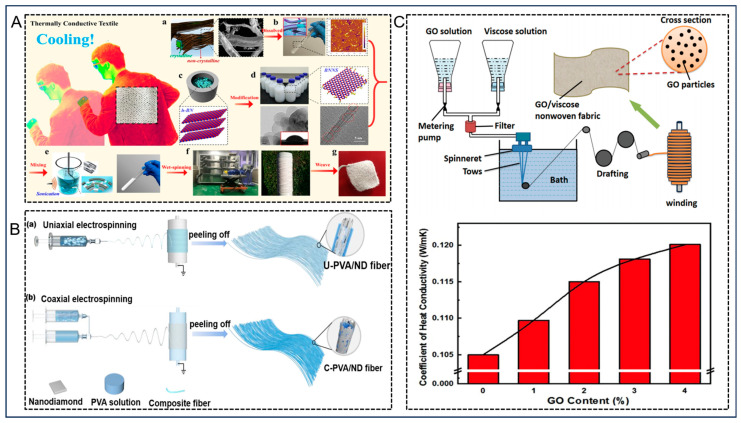
(**A**) Preparation of RCF/BNNS textiles. (**a**) The internal structure and morphology of untreated cotton linter pulp; (**b**) eco-friendly dissolution of cotton linter pulp for the production of wormlike cellulose with a one-dimensional structure; (**c**) the process of ball milling h-BN to achieve the synthesis of EOH-BNNS; (**d**) intense mechanical blending of cellulose and BNNS in alkali-containing aqueous solutions; (**e**) wet spinning of cellulose/BNNS concentrate blends for the preparation of RCF/BNNS fibers; (**f**) a visual representation of the woven RCF/BNNS textile; (**g**) the RCF/BNNS textiles. Reprinted with permission from Ref. [50]. Copyright 2019 American Chemical Society. (**B**) Flow chart of (**a**) uniaxial electrostatic spinning and (**b**) coaxial electrostatic spinning composite fibers; infrared image of temperature gradient between center and edge of LED chip at different times. Reprinted with permission from Ref. [53]. Copyright 2023 American Chemical Society. (**C**) The process of manufacturing GO-viscose fiber and nonwoven fabric; thermal conductivity of viscose fibers with varying levels of GO content. Reprinted with permission from Ref. [54]. Copyright 2020 Society of Plastics Engineers.

Recently, Wu et al. [50] incorporated boron nitride nanosheets functionalized with edge-selective hydroxyl groups (BNNS) with a high content of up to 60 wt% into a biodegradable cellulose/alkaline/urea aqueous solution. They successfully fabricated regenerated cellulose (RCF)/BNNS fibers using wet spinning technology (Figure 3A). BNNS, rather than carbon-based materials, were chosen as fillers because BNNS are inherently white, which makes textiles compatible with colored dyes without absorbing sunlight. The edge-selective hydroxylation of BNNS was designed through aqueous ball milling, which not only endowed them with good hydrophilicity but also maintained a high in-plane *λ* [51,52]. The axial thermal conductivity of the RCF/BNNS fiber reached 2.9 W m^−1^ K^−1^, which allowed it to dissipate the heat generated by the human body directly from the skin to the outer surface of the fabric through thermal conduction, thereby achieving a better personal cooling effect. In addition, the synergistic effect between excellent heat dissipation and good hygroscopicity can generate a better dynamic cooling effect for the wearer during certain physical activities. Its efficiency is even better than that of commercial wicking textiles such as cotton and RCF.

Lu et al. [53] at Guilin University of Technology have recently made progress in the development of high thermal conductivity thermal management materials that can reach thermal conductivity levels of 71.3 and 85.3 W m^−1^ K^−1^. As shown in Figure 3B, uniaxial and coaxial electrospinning methods were used to fabricate composite fiber films of uniaxial-polyvinyl alcohol/nanodiamond (U-PVA/ND) and coaxial-polyvinyl alcohol/nanodiamond (C-PVA/ND) with diverse microscopic morphologies. Both methods did not require complex pre-treatment procedures or the introduction of superfluous additives. According to the results, U-PVA/ND and C-PVA/ND composite fibers containing 60 wt% ND exhibited the *λ* of 71.3 and 85.3 W m^−1^ K^−1^, separately. These were 171.2 and 205.1 times higher than that of pure PVA fiber films. Furthermore, the C-PVA/ND composite fiber film exhibited a maximum thermal decomposition temperature of 364.3 °C and a volume resistivity of 2.29 × 10^15^ Ω·cm. This indicates that the composite fiber film had good thermal stability and electrical insulation. The results obtained from this experiment provided compelling evidence of the effectiveness of electrospinning technology in producing highly thermally conductive composites. Additionally, thermally conductive films can serve as the outer layer of electronic components to enhance heat dissipation and prolong their operational lifespan.

Furthermore, Jiang et al. [54] proposed a novel composite fiber material that leverages the benefits of graphene oxide (GO) and the fiber itself, and results in an outstanding photothermal conversion effect. This material was fabricated through wet spinning, and incorporating varying concentrations of GO into the viscose solution (Figure 3C). The photothermal conversion performance of different samples under near-infrared light was different. It is apparent that the temperature increase in the sample containing 4% GO was approximately 112 °C; however, that of the unadulterated viscose sample was about 50.8 °C. This further substantiates the fact that the incorporation of GO could enhance the rate of light absorption and photothermal conversion efficiency of viscose fibers, and lead to the GO viscose composites having better heat generation performance. The *λ* of the viscose fiber with 4% GO content reached 0.120 W m^−1^ K^−1^, and the *λ* of the control sample without GO was 0.105 W m^−1^ K^−1^. The results showed that the addition of GO could enhance the *λ* of the composite fiber material. The fiber is a promising material for photothermal conversion; it can be industrially produced and is widely used for solar energy collection and building heating.

The application of high thermal conductivity materials as coatings on the surface of the fibers is another way to increase the *λ* of the fibers [55,56,57,58]. Appropriate materials should be able to provide good thermal conductivity to the fibers without adversely affecting radiative heat dissipation. Carbonaceous materials, such as multi-walled carbon nanotubes (MWCNTs) and single-walled carbon nanotubes (SWCNTs), with great thermal conductivity have been used as thermally conductive coatings for fibers. According to reports, the *λ* of cotton fabric can be significantly improved by applying a resin containing MWCNTs as a coating on its surface. The presence of a coating with 11.1% MWCNTs resulted in a 78% increase in the *λ* of cotton textiles, and the fiber’s thermal conductivity increased by 1.5 times when the MWCNT content was increased to 50% [28]. The surface temperature of fibers coated with 50% MWCNTs was 3.9 °C lower than the surface temperature of fibers without any treatment at 50 °C.

**Figure 4:**
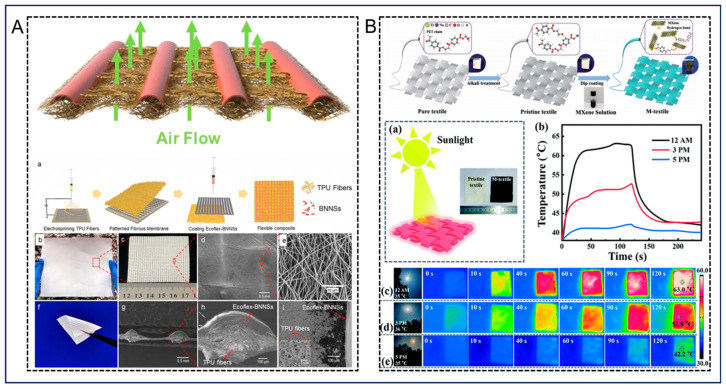
(**A**) Schematic representation of the permeability of composites based on patterned textile constructions; (**a**) the process of producing the composite material; (**b**,**c**) photograph of patterned electrostatically spun TPU fiber membrane; (**d**,**e**) SEM images of patterned electrostatically spun TPU fibers; (**f**) photograph of a fiber mesh sample covered with a layer of Ecoflex-BNNS; (**g**,**h**) cross-sectional SEM image of a fiber mesh covered with a layer of Ecoflex-BNNS; (**i**) the composite material is still breathable due to the presence of voids. Reprinted with permission from Ref. [59]. Copyright 2023 Nature Research. (**B**) Schematic diagram of M-textile preparation; (**a**) diagrammatic representation of the M-textile and untreated textile exposed to sunlight; (**b**) evolution of the temperature of M-textile-17.3 and the original textile exposed to sunlight for different periods of time; (**c**–**e**) infrared images of M-textile-17.3 and the original textile exposed to sunlight at different times. Reprinted with permission from Ref. [60]. Copyright 2020 The Royal Society of Chemistry.

Recently, Sun et al. [59] from Qingdao University reported a method to prepare a flexible thermally conductive composite fiber material with advanced thermal management capabilities. As shown in Figure 4A, the enhanced thermal conductivity was accomplished by applying a layer of boron nitride nanosheets (BNNS) with high thermal conductivity onto the grids of patterned electrospinning thermoplastic polyurethane (TPU) fibrous mats. The composites demonstrated a remarkable improvement in thermal conductivity while maintaining their original air permeability. Upon integration of the composite into a flexible device, the stable operating temperature experienced a notable decrease when compared to pure Ecoflex packaging. Surface temperature fluctuations were less than 0.5 °C during more than 2000 cycle tests. This work proposes a composite material that can be used in wearable electronic products with innovative thermal management capabilities and has promising development prospects.

As shown in Figure 4B, Liu et al. [60] employed a solution coating method in their study to cover the surface of robust, resilient, and abrasion-resistant polyethylene terephthalate (PET) fibers with MXene layers. This created a novel material called M-textiles. The synergistic effect of hydrogen bonding and physical riveting facilitated an intense connectivity between MXene and the fibers, and enabled an effective combination of the two. The specific structural diagram is illustrated in the figure below. These M-textiles maintained the inherent flexibility, comfort, lightweight, and breathability of a textile substrate. Furthermore, it demonstrated outstanding long-term stability in heating performance by encompassing dual-drive energy conversion, a wide range of temperatures, secure operating conditions, and rapid thermal response.

### 3.2. Thermally Conductive Fiber Materials for Electric Heating

Highly thermally conductive fibrous materials with fast-warming characteristics can be prepared by modifying or embedding electrically conductive materials onto the surface of thermally conductive textiles or their fibers using the Joule heating strategy of electrothermal conversion. Carbon-based materials (such as carbon nanotubes and graphene) [61], metallic nanomaterials [62,63] and conductive polymers [64] are viable candidates. In the presence of voltage, fibers generate a heating power in Joules that is suitable for warming the human body and can be used for personal thermal management. In addition to this, thermally conductive fiber materials for electric heating can be used in built-in heating systems for automotive seats, which not only provide sufficient heat for the driver, but also minimize the energy consumption of the vehicle’s interior [65].

**Figure 5:**
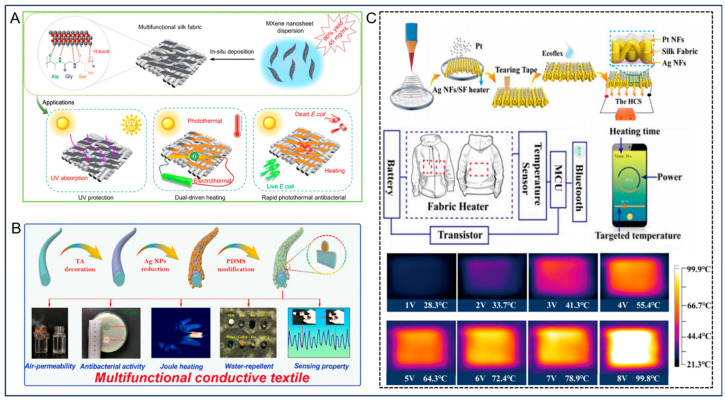
(**A**) Preparation of a high-yield and highly concentrated MXene dispersion, and multifunctional treatment of silk fabric. Reprinted with permission from Ref. [66]. Copyright 2021 American Chemical Society. (**B**) Schematic representation of the preparation of cotton/TA/Ag NPs/PDMS fibers with thermally conductive and superhydrophobic properties. Reprinted with permission from Ref. [69]. Copyright 2021 Springer. (**C**) Schematic diagram of the process for the preparation of sandwich-structured fiber textiles (Ag NFs/ fabric/Pt NFs); schematic circuit diagram of the smart thermal control system; infrared (IR) images and average temperature of the Ag NFs/SF heater (Rs = 25 Ω) at varying applied voltages ranging from 1 to 8 volts. Reprinted with permission from Ref. [64]. Copyright 2019 Institute of Physics Publishing.

Yan et al. [66] successfully prepared a high-yield (90%), highly concentrated (45 mg/mL) MXene dispersion with excellent uniformity with selective etching of Ti_3_AlC_2_ using in situ synthesis of HF. By utilizing van der Waals forces and hydrogen bonding (H-bond), the dip-coating method was utilized to deposit active MXene nanosheets in situ onto the fibers, achieving effective results (Figure 5A). The obtained fibrous silk textiles (MXene@silk) had good thermal and electrical conductivity and exhibited excellent electrical and thermal conversion properties. To assess the stability of the surface temperature in MXene@silk textiles, an investigation was conducted to observe the temporal temperature change under a constant voltage of 10V. After switching on the power supply, the temperature of the textiles increased rapidly to 60 °C and the temperature basically remained stable after 600 s. This demonstrates its long-term heating stability. The electric heating effect can be achieved at the fingers or wrists using MXene@silk heating materials; this proves that they can be used by those who need physiotherapy, rehabilitation, or abdominal and leg warmth. This highlights its enormous potential in personal thermal management applications.

In the past few years, there has been growing global interest among researchers and industrial companies in electrically conductive yarns containing metal fibers. These yarns are utilized in the production of knitted or woven fabrics for various applications including personal protection, healthcare, defense, communication, and electronic automation. Silver nanoparticles are considered an important candidate for the preparation of wearable electronic devices due to their outstanding electrical and thermal conductivity [67,68], and mechanical flexibility. Ensuring stable adhesion of silver nanoparticles on fiber surfaces is a key focus in designing and preparing multifunctional fiber textiles. Guo et al. [69] prepared a multifunctional thermally conductive and electrically conductive fiber (cotton/TA/Ag NPs/PDMS) with super-hydrophobicity, personal thermal management, and human movement tracking by using tannic acid (TA) coating for the in situ synthesis of silver nanoparticle (Ag NPs) on cotton fibers followed by modification treatment with polydimethylsiloxane (PDMS) (Figure 5B). The excellent thermal conductivity, electrical conductivity and flexibility of cotton/TA/Ag NPs/PDMS fibers make them a potential candidate material for wearable heating devices for personal thermal management. The cotton/TA/Ag NPs/PDMS fiber textiles not only maintained the intrinsic elasticity, comfort, and breathability of cotton textiles, but also exhibited antimicrobial properties, rapid thermal responsiveness, and exceptional long-term stability in heating performance. It is anticipated that this can be utilized in thermal regulation and human motion tracking; this offers a new direction for the exploration of next-generation lightweight, portable, and wearable electronic fibers and textiles.

Textronics is regarded as one of the cutting-edge fields of scientific research [70]. Huang et al. [64] reported a new sandwich-like configuration of fiber textiles (Ag NFs/fabric/Pt NFs). The Ag nanofiber network attached to the wearable fiber textiles can be used as a heater, while the Pt nanofiber array functions as a temperature sensor. The high thermal conductivity and thermal stability of this sandwich-structure textile and the accuracy of the Pt nanofibers as temperature sensors showed that there is potential for it to be used with remote interactive control via smartphones. Figure 5C illustrates a schematic representation of a heating control system (HCS) utilizing a fiber fabric, which consists of a heater, temperature sensor, microcontroller unit (MCU), and Bluetooth module. The entire system could be remotely regulated and observed via a smartphone, and the temperature configured through a mobile application (APP). The digital output signal from the smartphone governed the energy module’s output, which could be utilized to regulate the temperatures of the Ag NFs heaters. The complete utilization of smart temperature controllers has not yet been fully demonstrated and there are still areas that need improvement and attention, such as laser patterning that can provide high accuracy and repeatability in the geometry and electrical characteristics of heater-sensor structures [71]. However, this study offers valuable inspiration for potential avenues of research in future directions for integrated and interactive advanced textiles.

### 3.3. Thermal Radiation Conductive Fiber Materials

The development of personal thermal management technology is dependent on the fundamental heat transfer theories; specifically, conduction, convection, and radiation. Over 40% of the heat exchange between the human body and the environment comes from infrared thermal radiation [72,73]. Infrared radiation within the wavelength range of 8–13 μm, known as long-wave infrared (LWIR), is referred to as the “atmospheric window”. Via this window, excess heat can dissipate to a cooler outer space [74,75,76,77]. Therefore, the key focus in the current development of personal thermal management materials is the exploration of heat-conductive fiber materials that can regulate LWIR thermal radiation around the human body. Passive thermal management has been demonstrated to achieve diverse thermal regulation without the need for energy consumption through the reflective, emissive, and transmissive properties of materials. This represents a significant breakthrough in moving away from traditional heating and cooling techniques [78,79,80,81,82]. Fiber textiles based on radiative thermal control have broad prospects [82,83,84].

**Figure 6:**
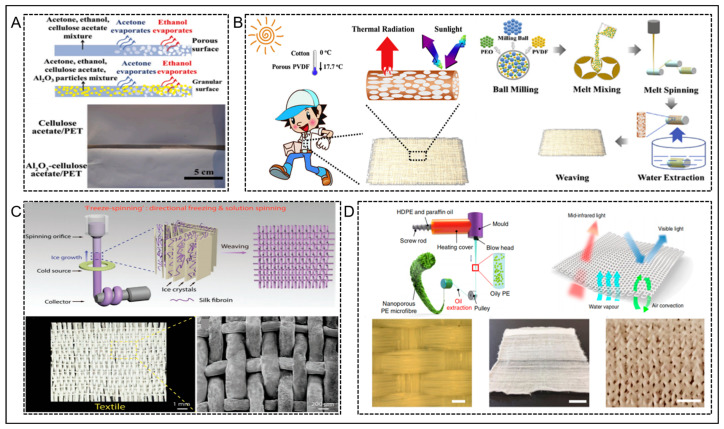
(**A**) Schematic diagram of the phase transition process where HDPE refers to high-density polyethylene; a captured image depicting the PET substrate coated with cellulose acetate and Al_2_O_3_-cellulose acetate in the presence of sunlight. Reprinted with permission from Ref. [85]. Copyright 2020 Elsevier. (**B**) Novel porous polyvinylidene fluoride (PVDF) fibers and their textiles for passive cooling of the human body; schematic diagram of the PVDF fiber preparation process. Reprinted with permission from Ref. [87]. Copyright 2020 Elsevier. (**C**) Schematic illustration of the “freeze spinning” technology; optical and scanning electron microscope images of typical bionic porous fiber woven textiles. The fiber diameter is around 200 μm. Reprinted with permission from Ref. [90]. Copyright 2018 Wiley-VCH. (**D**) Schematic illustration of the preparation process for the nanoPE microfiber; schematic illustration of the nanoPE textiles; the nanoPE textiles with high and medium infrared transparency, visible light opacity and good wearing comfort; enlarged view showing woven nanoPE textile structure; a picture of a knitted nanoPE textile; a magnified image of the knitted nanoPE textile revealing its intricate knitted structure. Reprinted with permission from Ref. [86]. Copyright 2018 Springer Nature.

Wei et al. [85] prepared an Al_2_O_3_-cellulose acetate fiber textiles that could enhance the material’s thermal conductivity and solar reflectivity (Figure 6A). Cellulose acetate has a high radiation rate in the long-wave infrared region; this promoted effective longwave infrared radiation (2.5–25 μm). Meanwhile, Al_2_O_3_ owns a high thermal conductivity (30–40 W m^−1^ K^−1^), which could improve solar reflectivity. By coating Al_2_O_3_-cellulose acetate, the solar reflectance of the fiber textiles was significantly increased from 62.6% to 80.1%. Cooling performance experiments showed that the modified textiles could reduce simulated human skin temperature by 2.3–8 °C compared with the unmodified control samples. In addition, in real cooling performance experiments, the modified T-shirt prevented overheating of actual human skin; this corresponds to a reduction of 1.9–3.3 °C in the inner surface temperature of the textiles. This study provides a new idea and methodology for the purpose of designing and synthesizing radiatively conductive fiber textiles for personal thermal management.

Cui et al. [86] prepared nano polyethylene (PE) fibers through a fiber extrusion procedure, and then obtained nano polyethylene (nanoPE) textile fibers through weaving (Figure 6D). The nano polyethylene fiber textiles not only maintained high transmittance to infrared radiation and opacity to visible light, but also improved abrasion resistance. In comparison to cotton textiles of equivalent thickness that are available commercially, the nano-fiber textiles had the ability to reduce human skin temperature by 2.3 °C; which indicates its excellent heat dissipation performance and cooling ability. Mechanical tests demonstrated that the nano polyethylene fibers also possessed good abrasion resistance. In addition, the polyethylene nanofibers and textiles can be prepared on a large scale by industrial machines, offering broad prospects for development.

Recently, Song et al. [87] prepared polyvinylidene fluoride (PVDF) radiative cooling fibers with a porous structure using an industrial melt spinning method combined with a two-phase melt separation pore formation technique, as shown in Figure 6B. The pore morphology of the fibers was regulated by changing the ratio of PVDF and polyethylene oxide (PEO) in the PVDF/PEO blends, and the addition of a small amount of PEO facilitated the formation of porous structures with smaller sizes. The introduction of the porous structure enhanced the light-scattering intensity of the PVDF porous fibers and improved the sunlight shielding ability of the PVDF porous fibers and their textiles. The optimized PVDF porous fibers and their textiles exhibited an absorption/emission rate of up to 94.5% in the atmospheric window range (8–13 μm), while the absorption/emission rate in the sunlight band was reduced to a minimum, and the reflectivity was significantly improved; this increased the heat radiation release and shielding the heat absorption. Under direct sunlight exposure, the temperature of simulated skin covered by PVDF porous fiber textiles was reduced by 17.7 °C compared with the naked state, which was four times that of traditional PVDF fiber textiles and two times that of traditional cotton textiles. This greatly improves thermal comfort for the human body.

**Figure 7:**
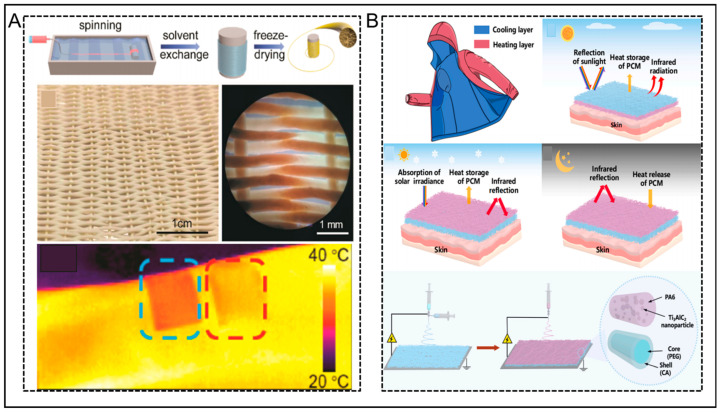
(**A**) Schematic diagram of KNF aerogel fiber preparation; photographs of textiles woven from aerogel fibers; polarized light microscope images of aerogel textiles; infrared photographs of aerogel textiles and cotton textiles tested for room temperature insulation properties. Reprinted with permission from Ref. [89]. Copyright 2019 American Chemical Society. (**B**) Structural design and principles of all-weather personal thermal management textiles; illustration depicting the schematic process of preparing TAWT. Reprinted with permission from Ref. [24]. Copyright 2023 Elsevier.

Aerogels exhibit significant potential as optimal thermal insulation options because of their low density, extensive surface area, and porous nature. They can simultaneously inhibit heat conduction, convection, and radiation [88]. In the study by Liu et al. [89] depicted in the Figure 7A, bulky aerogels were transformed into one-dimensional aerogel fibers for personal thermal management in harsh environments. They made Kevlar (KNF) aerogel into aerogel fibers by wet spinning, and they were then woven into textiles with excellent mechanical properties and extraordinary thermal insulation. When the aerogel fabric was applied to the human arm, it exhibited lower infrared temperatures compared to cotton fabric. This validates its superior personal insulation potential in high-temperature environments.

Bai et al. [90] were inspired by polar bear hair and developed a large-scale production method for polar bear biomimetic fibers using a technique called “freeze spinning” (Figure 6C). These biomimetic fibers have an aligned porous microstructure with a porosity as high as 87%, which contributes to their excellent thermal insulation properties. They then wove the bionic fibers into textiles and conducted experiments using infrared and covering rabbits with the fabric. The biomimetic textiles showed superior insulation performance compared to commercial polyester fibers of equivalent thickness, as their temperature approached the ambient background temperature. Additionally, the biomimetic fabric performed well within the temperature range of −10–40 °C, demonstrating its significant potential for various applications.

Recently, a novel all-weather personal thermal management (PCM) textile, TAWT, was developed by Fan et al. [24]. This integrated radiative cooling, solar heating, thermal storage, and phase change release into a triple-mode system (Figure 7B). Their approach involved the integration of two layers with distinct optical and thermal characteristics into a single textile. By employing coaxial electrospinning, fibers with a core–sheath structure were prepared using cellulose acetate (CA) as the sheath layer and polyethylene glycol (PEG) as the core layer. The fiber was used as the cooling layer. The heating layer consisted of polyamide (PA6) fibers that were infused with Ti_3_AlC_2_ (TAC) nanoparticles. In cooling mode, TAWT achieved a solar reflectance of 96.6% and a mid-infrared emissivity (MIR, 7–14 μm) of 93.2%. The cooling layer was oriented towards the environment to reduce the absorption of solar radiation to a minimum and enhance radiative cooling. The thermal storage function of the PEG provided an external cooling function that compensated for the lack of radiative cooling capacity in hot and humid weather. Compared to bare skin and traditional textiles, the temperature was reduced by 13.1 °C and 9.2 °C, respectively. In the heating mode, TAWT exhibited a solar absorptance of 82.0% and a low emissivity in the far-infrared range (67.8%), which could prevent overcooling. During cold days, the absorption of solar radiation provided warmth to the body, while the low emissivity prevented heat loss. the PEG also stored excess solar energy as latent heat, which could be released when the ambient temperature decreases to keep the body warm. In addition to its thermal management features, it also demonstrated excellent durability. With these favorable characteristics, TAWT provides advanced ideas and methods for the development of multifunctional textiles.

### 3.4. PCM Thermally Conductive Fiber Materials

Phase Change Materials (PCMs) refer to substances that supply latent heat through a change in their physical state without a change in temperature. As a smart material, PCMs have the capability to absorb and retain thermal energy in the form of latent heat, subsequently releasing the stored heat at a specific critical temperature during phase transition. This confers benefits such as high heat storage density, economical cost, and strong chemical stability. Organic PCMs, notably paraffin and polyethylene glycol (PEG), have garnered considerable interest owing to their exceptional cyclic stability, non-toxic nature, and high latent heat capacity [91,92]. Owing to the high heat capacity and constant temperature characteristics of phase change materials (PCMs), integrating existing PCMs into fabric allows for the uniform distribution of PCMs within the fabric, thereby enhancing thermal comfort and exhibiting broad application prospects in the field of thermal management [93,94,95]. However, poor thermal conductivity, leakage issues at high temperatures, and lack of flexibility significantly restrict their potential application [96,97,98]. In addition, a large mass of PCM material, on which the heat capacity then depends, cannot be incorporated into textile materials. This means that, due to the low mass of PCM, the effect on the thermal properties is low. Therefore, the preparation of phase change fiber materials is not easy. Meanwhile, in order to provide thermal comfort with PCM fiber textiles, the phase change temperature of PCM should be controlled in the range of 18–36 °C. For example, n-decane, n-heptane, n-eicosane, and n-octadecane have been applied in the field of smart thermal conductivity [99,100]. Various methods such as coaxial electrostatic spinning, microencapsulation, and polymer backbone coating have been explored as solutions to the leakage problem [101].

**Figure 8:**
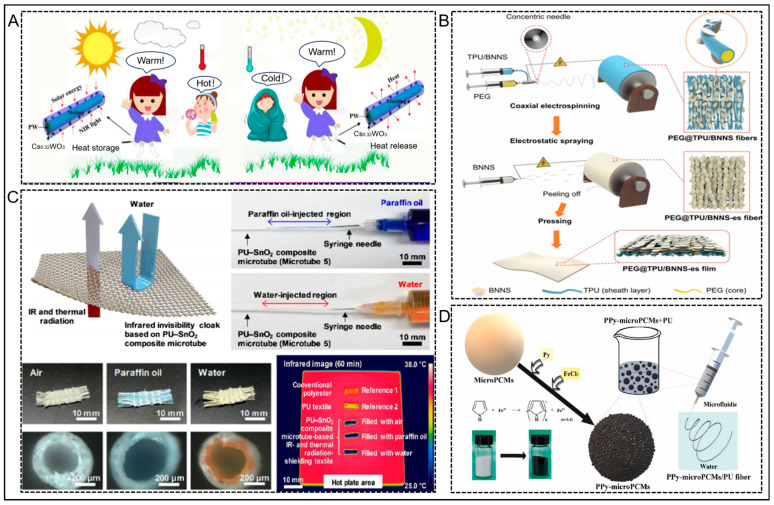
(**A**) Diagrammatic representation of the intelligent textile utilizing PW-encapsulated core–sheath structure fibers designed for integration into garments for human use. Reprinted with permission from Ref. [104]. Copyright 2019 Elsevier. (**B**) Illustration of the procedure for fabricating the PEG@TPU/BNNS-es nanocomposite films. Reprinted with permission from Ref. [105]. Copyright 2023 Springer Nature. (**C**) Diagram demonstrating a textile with infrared and thermal radiation shielding, as well as water-repellent characteristics; injecting paraffin oil or water into the PU-SnO_2_ composite microtube with a syringe; infrared thermal pictures of the textiles with shielding properties against IR and thermal radiation; optical image of the textile with shielding properties against IR and thermal radiation, along with a cross-sectional view of the microtube. Reprinted with permission from Ref. [106]. Copyright 2012 Elsevier. (**D**) Schematic representation of the preparation of the photothermal PPy-microPCMs/PU fibers. Reprinted with permission from Ref. [107]. Copyright 2021 Elsevier.

The coaxial electrostatic spinning method allows the preparation of fibers with a core–sheath structure from separated channels of diverse pathways [102,103]. For example, the inner channel is filled with a solution of PCM, while a polymethyl methacrylate (PMMA) or PMMA-CNT co-solution is injected into the outer channel. This results in the formation of nanofibers with a core-shell structure. consequently, the PCM can be encapsulated within the core–sheath structure, thereby solving the issue of leakage. Lu et al. [104] prepared smart fiber textiles with a core–sheath structure by using coaxial electrostatic spinning method, with paraffin wax (PW) as the core layer and polyacrylonitrile (PAN) as the sheath layer, to overcome the leakage problem of PW (Figure 8A). The resulting fibers had a tubular shape, 190 nm in diameter. In order to improve the heat-absorbing properties of the fibers, they further supplemented hexagonal cesium tungsten bronze (Cs_0.32_WO_3_), which had a good near-infrared (NIR) absorption capacity, to the PAN material; this resulted in the PCM-based hybrid nanofibers. Infrared experiments demonstrated that, compared to pure PW textiles, PCM-based nanofiber textiles exhibited excellent heat absorption performance and showed great potential for thermal regulation. Additionally, after 500 cycles of heating and cooling, the latent heat of the smart fiber textiles remained almost unchanged, indicating great stability. This study offers valuable insights for the advancement of smart fiber textiles.

In contrast, Huang et al. from Shanghai Jiao Tong University [105] used a strategy combining coaxial electrostatic spinning and electrostatic painting to prepare a high thermal conductivity phase change nanocomposite fiber with a core–sheath structure and a highly oriented boron nitride nanosheet (BNNS) interconnected network structure; this was further fabricated into a film called PCN (Figure 8B). The PCNs exhibit a core–sheath configuration, with the polyethylene glycol (PEG) serving as the core and the sheath composed of thermoplastic polyurethane (TPU) nanocomposites reinforced with BNNS. The introduction of the sheath layer made of flexible polymer not only effectively prevented the leakage of the phase change component but also endowed the phase change composite material with good flexibility. Simultaneously, the high orientation of the boron nitride network enhanced the thermal conductivity efficiency, significantly improving the *λ* of the phase change composite material, while still maintaining its high heat capacity characteristics. Analysis revealed that the core–sheath structure exhibited excellent encapsulation, and prevented leakage of the phase change component and endowed the PCN with outstanding flexibility, while maintaining a high latent heat. The highly oriented BNNS interconnected network enabled the PCN to demonstrate an ultra-high in-plane *λ* (28.3 W m^−1^ K^−1^). In addition, the PCN possessed excellent fire resistance and electrical insulation properties, showing great potential for utilization in thermal management of 5G base stations.

Through technical means, phase change materials can be encapsulated in a polymer shell to form micron or nanometer-sized microcapsules (i.e., phase change microcapsules). PCMs microencapsulated in shell structures not only overcome the leakage problem of the PCM, they also avoid the interaction between PCMs and the external environment, increase the heat transfer surface area, effectively improve the *λ*, and localize the phase change process. This solves the issue of significant volume changes occurring during the phase change process [108]. Microencapsulation of phase change materials can be achieved through mature techniques utilized in the textile industry; for example, coating, filling, lamination, and printing. Additionally, fibers with a hollow structure are ideal materials to act as tiny containers for phase change materials. As shown in Figure 8C, Ju et al. [106] prepared polyurethane (PU)-tin dioxide (SnO_2_) composite hollow fibers using wet spinning, and obtained a thermal radiation shielding fiber textile. PU-SnO_2_ solution was obtained by dissolving different contents of tin chloride dihydrate (0.5 g, 0.7 g, 0.8 g, 0.9 g, 1.0 g, 1.1 g, and 1.2 g, respectively) in equal amounts of 12 wt% PU solution, and then PU-SnO_2_ composite hollow fibers were prepared by wet spinning. The diameter of the PU-SnO_2_ composite hollow fibers was approximately 200 μm. The hydrophilic urethane bonds of the PU polymer enable the easy substitution of the solvents tetrahydrofuran (THF) and dimethylformamide (DMF) in the PU solution with water. Due to the hydrophilicity of both the PU matrix and embedded SnO_2_, the exchange between DMF and THF solvents in the PU-SnO_2_ solution with water was relatively straightforward, even during the wet spinning process of PU-SnO_2_ composite hollow fibers. Subsequently, the PU-SnO_2_ composite hollow fibers were formed by phase separation. In addition, filling the core of PU-SnO_2_ composite fibers with heat-absorbing materials (such as water and paraffin) instead of air can lead to enhanced infrared and thermal radiation shielding properties, indicating promising prospects for broad development [109].

Hu et al. [107] developed flexible photothermal phase change fibers with high thermal conductivity by compositing polyurethane (PU) and commercial photothermal phase change microcapsules (microPCMs) modified with polypyrrole (PPy) using a straightforward wet spinning technique (Figure 8D). The findings indicated that the photothermal storage efficiency of the microPCMs was as high as 94.03% at a PPy encapsulation of only 1.91 wt%. Meanwhile, the microPCMs/PU composite fibers maintained high elasticity (365% elongation) with a microPCMs load of 79.45 wt%.

## 4. Summary and Outlook

As society grows and develops, heat dissipation has become a key issue for high-power-density electronic equipment and devices [110,111,112,113,114,115]. Moreover, there is an increasing demand for thermal comfort and concepts such as PTM have gained prominence. This has led to a diversified and specific demand for thermally conductive materials, particularly those with efficient thermal management properties. Among these, smart thermally conductive fiber materials, known for their high thermal conductivity and smart response properties, are garnering increasing attention. This review explored various innovative and logical technologies for effective thermal management. Fiber textiles that facilitate efficient thermal management are crucial for energy conservation, heat dissipation, and thermal regulation. This paper briefly reviews emerging thermally conductive fiber materials and their applications, based on various heat transfer mechanisms, and classifies them based on their preparation methods and principles. In the realm of PTM, addressing heat transfer and dispersion from the human body is crucial. Active cooling can be achieved by integrating fibers with highly thermally conductive fillers. Thermally conductive fiber textiles with passive heating characteristics (photothermal heating) and active heating characteristics (electrical heating) can be used for insulation. In addition, fibers combined with phase change materials and smart fiber textiles based on thermal radiation can provide a dual response of cooling and warming in varying environments. Phase change materials, which are capable of storing and converting latent heat, offer improved temperature regulation for the human body, particularly in response to drastic outdoor weather changes. In the field of thermal management of electronic devices, thermally conductive fiber materials can be further processed into thin films. Their design flexibility in terms of dimension and structure allows for good plasticity and adaptability. Furthermore, their excellent thermal conductivity makes these materials suitable for heat dissipation in electronic components. They offer extensive application prospects in the thermal management of high-power-density electrical equipment and emerging electronic devices.

From this review, it is evident that smart thermally conductive fiber materials have undergone rapid development. However, addressing the following key challenges remains imperative:

(1) Some materials designed for PTM have difficulty striking a balance between thermal management performance and wearing comfort, such as breathability, moisture absorption, and softness of fiber textiles [116,117].
(2) The manufacturing process of thermal management fibers combining advanced materials is complex and costly; this prevents them from being widely available on a large scale.
(3) Ensuring the long-term stability and durability of smart thermally conductive fiber materials is crucial. This includes maintaining their thermal conductivity properties even after repeated use and exposure to various environmental conditions.
(4) There is a need for a comprehensive integrated evaluation system to evaluate the thermal management capabilities of smart thermally conductive fiber materials effectively.
(5) Requirements with respect to the temperature threshold of phase change materials are often very stringent, and the transition point of many materials considerably deviates from room temperature, restricting their application scope.
(6) Smart thermally conductive fiber materials should be compatible and easily integrated into existing products and systems, such as clothing, bedding, and electronic devices. This requires consideration of factors such as compatibility with various fabrication techniques and resilience to mechanical stresses.
(7) The regulation mechanism of smart thermally conductive fiber materials still needs to be studied in depth; this includes their thermal conductivity under different microstructures.
(8) The materials must meet safety standards and ensure user comfort, encompassing biocompatibility, non-toxicity, breathability, and flexibility for a comfortable and safe user experience.
(9) Considering the growing concern for environmental sustainability, it is essential to assess the environmental impact of these materials throughout their lifecycle, from production to disposal. Developing eco-friendly alternatives and recycling methods is crucial for sustainable practices.

In conclusion, the thermal management performance of fibers can be improved by introducing new materials and modifying their structure. To develop new thermally conductive fiber materials, researchers should consider combining various methods or exploring new techniques to maintain or improve the *λ* of the fiber, while preserving its inherent properties such as flexibility. These innovations offer fresh perspectives and methodologies for future development in this field.

## Data Availability

Not applicable.
